# Beating Darwin-Bragg losses in lab-based ultrafast x-ray experiments

**DOI:** 10.1063/1.4978742

**Published:** 2017-03-24

**Authors:** Wilfred K. Fullagar, Jens Uhlig, Ujjwal Mandal, Dharmalingam Kurunthu, Amal El Nahhas, Hideyuki Tatsuno, Alireza Honarfar, Fredrik Parnefjord Gustafsson, Villy Sundström, Mikko R. J. Palosaari, Kimmo M. Kinnunen, Ilari J. Maasilta, Luis Miaja-Avila, Galen C. O'Neil, Young Il Joe, Daniel S. Swetz, Joel N. Ullom

**Affiliations:** 1 Department of Chemical Physics, Lund University, Box 124, Lund SE-22100, Sweden; 2 Department of Applied Mathematics, RSPE, Australian National University, Canberra, ACT 2601, Australia; 3 Department of Chemistry, The University of Burdwan, Golapbag, Burdwan 713104, WB, India; 4 Department of Physics, Chemistry and Biology (IFM), Linköping University, 58183 Linköping, Sweden; 5 Nanoscience Center, Department of Physics, University of Jyväskylä, P.O. Box 35, FI-40014 Jyväskylä, Finland; 6 National Institute of Standards and Technology, Boulder, Colorado 80305, USA

## Abstract

The use of low temperature thermal detectors for avoiding Darwin-Bragg losses in lab-based ultrafast experiments has begun. An outline of the background of this new development is offered, showing the relevant history and initiative taken by this work.

## INTRODUCTION: A CENTRAL REQUIREMENT

Crystallographic Darwin-Bragg losses are a leading cause of low detectable flux in X-ray spectroscopy studies, which study the energy exchange (*ω*) of photons of incident energy *E* with samples. The frustration is most acute where high *ω*-resolution is needed in broadband measurements. The corresponding loss is typically a factor ∼10^−5^, according to the Darwin spectral (or angular) acceptance width, with the possibility of greater throughput if lower resolution will suffice, but in any case relative to the spectral region of interest (ROI).^1,2^ A huge variety of conventional Bragg-based spectroscopic arrangements exists, whereby samples, monochromators, analysers, and instrument topology each have more or less bearing on diffraction widths and efficiency. In that collective sense, spectral ROIs are both arbitrary and potentially very broad. For X-ray spectroscopy then, accurate energy-resolving approaches are sought that can accommodate diverse needs without incurring Darwin-Bragg losses at any level.

In another very broad class of X-ray techniques, diffraction-based measurements from substantial volumes of reciprocal (momentum transfer, *Q*-) space are sought that correspond to elastic scatter (*ω* = 0). In these, a basic requirement is again the knowledge of the photon energy. This is seen in the relationships *Q* = |*k_f_ − k_i_*| = *2π/d = (4π/λ)*sin *θ* = *(4πE/hc)*sin *θ* for initial and final momentum vectors *k_i_* and *k_f_*, diffraction angle *2θ*, and correlations of size *d* in real space. Note that directions of *k_i_* and *k_f_* are both defined by knowledge of the places where generation, scatter, and detection occur. For this Bragg diffraction situation, broadband and/or high divergence (i.e., *reduced* brilliance) sources can address large volumes of reciprocal space for any particular sample orientation (e.g., in individual radiation shots),^3–6^ while extreme brilliance sources cannot. The variables *E* and *θ* contribute to *Q* deviation according to *dQ = (∂Q/∂θ)dθ + (∂Q/∂E)dE*. Here the first term shows the need for a low angular uncertainty in diffraction measurements. Traditional divergent and convergent beam geometries typically use the common angle theorem of cyclic quadrilaterals to avoid losses while accurately knowing *θ* in diffraction setups. Different diffraction measurements have different needs for Q-resolution, such that geometric design compromises are generally possible. More conveniently for pump-probe diffraction measurements, the incident beam may be collimated, as per the laser wakefield-based^7–9^ and FemtoMAX^10–12^ investments made as part of this work.^5^ But in any case, the second factor again shows the simultaneous need to accurately know photon energies in polychromatic diffraction contexts, to enable *Q-*resolution. In limited flux situations, diffracted photon energies must be established efficiently.

More generally, momentum changes (*Q*) and energy changes (*ω*) are not mutually exclusive. In this broader sense, spectroscopy and diffraction together constitute the scattering function *S(Q,ω)* containing all that can be learned about the sample from scattered radiation. *S(Q,ω)* relates directly to the sample's structure correlations and their dynamics. This comes about *via* a Fourier transform in space and time, which connects *S(Q,ω)* to its time-dependent pair correlation function *G(d,t*).^13–16^ It applies to neutrons and other de Broglie wave quanta as well as X-rays.^17^ The combination of conceptual need and practical constraint described above often motivates the use of polychromatic radiation; but then the knowledge of the energy of quanta is effectively a requirement.

Brilliant sources require a sequential approach to mapping out *S(Q,ω)*. That is a problem for ultrafast work, since samples cannot be reoriented within a single shot. Together with an optical excitation pulse, a single shot can be all it takes to damage or destroy a sample. Yet a representative sampling of *S(Q,ω)* space is essential to enable the Fourier transform to *G(d,t)*. Neutron work addressed the latter need around low brilliance sources aided by time of flight (TOF) detection methods. Building on TOF neutron structural dynamics studies, this work introduced low temperature thermal detectors to the lab-based ultrafast laser-driven X-ray field, for those reasons.

The manuscript is structured as follows. The “Constraints of Brilliance” section gives a description of the sample structure and spectroscopy features whose observation is lost in ultrabrief collimated monochromatic radiation shots. It is followed by a short discussion of the foundational role that polychromatic neutron time of flight approaches continue to play in molecular structural dynamics contexts, in which they avoid the same losses. Then, the underpinning thermodynamic approach and considerations required by energy dispersive and low temperature thermal detectors to achieve the same outcomes for X-rays is addressed. We close with a brief prospectus and indication of the current state of developments for the first couplings of lab-based ultrafast laser driven X-ray sources with such detectors, which this collaboration has initiated on two continents.

## CONSTRAINTS OF BRILLIANCE

**FIG. 1.**
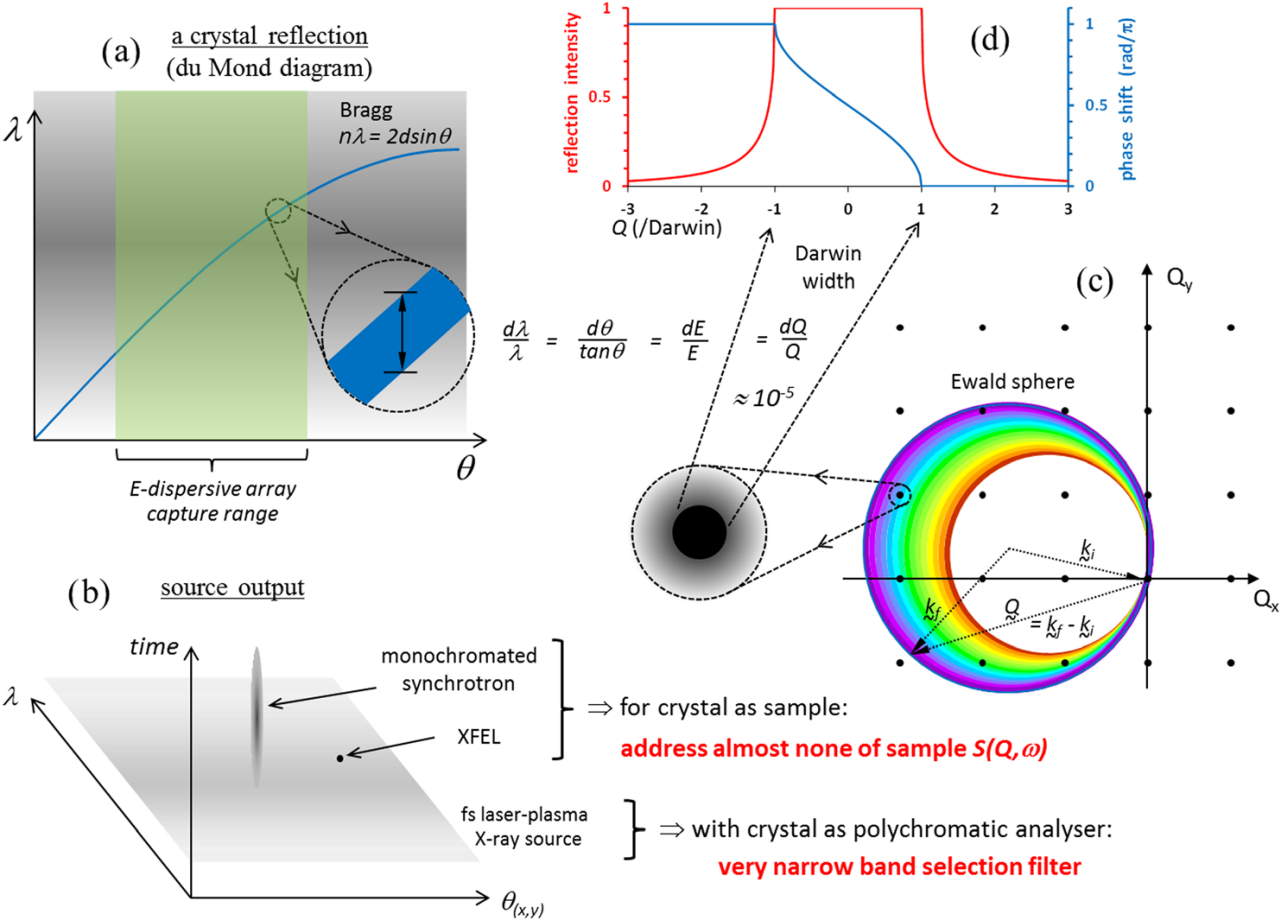
Energy dispersive X-ray array and neutron TOF detectors avoid Darwin losses, opening efficient and comprehensive views of samples' *Q*- and *ω*-space. Bragg scattering traces a sinusoid line on a du Mond diagram (panel (a)). Phase space occupancy of radiation sources can be roughly shown by its extension with a time axis (panel (b)). In panel (a) an energy dispersive array captures the highlighted angle range allowing efficient spectroscopy (*ω*-studies),^18,19,139,142,163,164^ while Bragg diffraction analyses transmit only what is under the width of the sinusoid line. The Bragg equation can also be shown in momentum transfer space (Q=(4π/λ) sin θ=2π/d; panel (c)), where crystal reflections appear as dots. The geometrical size of line and dots in panels (a) and (c) correspond to the Darwin width^68^ in the limiting case of infinite ideal crystals (panel (d)).^1,2^ Measuring structural dynamics *via* scattering problems requires representative observation of a sample's *Q- a*nd *ω*-space intensity features, impossible when probing structured samples with just one membrane-thin monochromatic Ewald sphere. Lowering brilliance by increasing divergence or bandwidth permits parallel collection of entire volumes of *Q*-space intensity features in single shots; the Laue sketch in panel (c) diffracts different colours of a small divergence but polychromatic beam.^3,4,96,196^

Figure 1 offers a graphical representation of the situation discussed in the following paragraphs. A metric often applied to X-ray sources is brilliance. It is the number of photons produced, normalised by their spectral bandwidth, the time interval in which they are produced, the physical dimensions of the source, and their angular divergence from it. Minimising the normalisation terms without overly compromising the total number of photons is often desirable, and increases the metric. Doing so accommodates the narrow acceptance conditions of diffractive and refractive X-ray optics, and the need to address small samples. It also makes spatial coherence effects more easily observable in the absence of energy resolving detectors that could otherwise determine structural effects *via* polychromatic *Q*-measurements. The resulting developments have led from low through moderate to high and extreme brilliance sources. In Figure 1, the temporal extension of the du Mond diagram roughly sketches a comparison of single pulses from three sources in terms of their bandwidth, divergence, and pulse duration contributions to single-shot brilliance. Another potential normalising parameter likely to become of increasing interest is the degree of polarisation (linear or circular), for examinations of spin and topological systems. Other factors also critically affect the practical usefulness of a source and could be incorporated in a metric. A partial list would include: repetition rate and temporal structure; broadband energy span; capacity for integration with other contemporary work; routine accessibility; safety; and affordability. The incorporation of low-T thermal X-ray photon detectors can go a very long way towards enabling X-ray structural dynamics autonomy in groups that have investments in moderate and high power ultrafast laser technologies and the desire to use hard radiations for ultrafast chemical examinations. It is the reason for our investments in this lab-based development, subsequent to and in parallel with major facilities, which can also benefit from them. The advantage comes about by these detectors' removal of Darwin losses suffered by Bragg diffraction instrument topologies, which was the only way to foresee adequate X-ray progress during its first century. In 2006, a confluence of the rationales described in this work was used to motivate the introduction of low temperature thermal detectors in lab-based ultrafast X-ray contexts. Their combination achieved “first light” in 2010 through the international collaboration represented in this work.^18,19^

When tied to the use of a high brilliance source for monochromatic photocrystallography,^20^ compromises are either in the temporal domain,^21,22^ or by measurements that necessitate vast serial acquisitions of individual shots to ensure due representation of every sample orientation with respect to its symmetry. This may be done stochastically, requiring extensive computational reconstruction.^23^ Due to their tight monochromation and power, X-ray free electron laser (XFEL) beams can also be used for spectroscopy when different energies are presented in different shots, thereby effectively sampling *ω*-space.^24^ Temporally chirped schemes are another possibility.^25^ Such workarounds are achievable in the cited *S(Q,ω)* explorations by applying major facility resources. Yet as earlier demonstrated,^3,4,6,13,25,26^ it is true that where the need is a *sample examination in S(Q,ω) space*, then the tightly squeezed parametric phase space of brilliant sources can be a severe handicap. Monochromatic interests are on one hand a concession to the challenge of photon energy resolvability in the X-ray field, which we deal with here. On the other hand they have meanwhile opened the door to interesting approaches appealing to speckle correlation in spatially coherent sources, phase conjugation/time reversal, and other suggestions and demonstrations that often end up motivating polychromatic approaches.^25,27–31^

There is a problem of phase preservation for X-rays in detectors, that is accepted in our broadband approaches and which does not apply at optical energies. Physically, the loss of phase information in detectors corresponds to the inability to preserve the spatial beat structure of interference fringes^32^ for short wavelength quanta at high *Q*. For high resolution this needs a fringe fidelity on vanishingly small length scales (recall *Q = 2π/d*); however, the finer the fringes, the more they are smeared by the relatively huge physical dimensions of observable radiation event phenomena.^33–35^ This constraint was seemingly recognised by the Braggs, noting text by Wilson^36^ and relevant for their development of optically reconstructed X-ray diffraction approaches.^37–40^ Preservation of X-ray phase in detectors at high *Q* corresponding to molecular distances may never find a generally practical solution, while source coherence developments do not change that situation. At optical energies, Ewald spheres cannot access molecular dimensions (Figure 1(c)), but optical wavelengths are long and photon energies low compared to X-rays, so the interference fringe fidelity is preservable. That has been the basis for Lippmann colour photography,^41–45^ holography^32,46–48^ and optical phase conjugation,^49–53^ leading among other things^54,55^ to the coherent multidimensional spectroscopies^56^ now widely practiced in ultrafast laser labs.^57–61^ Optical laser technologies have stimulated efforts to allow comparable effects at higher photon energies, in particular, X-rays,^62–65^ where matter and its ultrafast molecular movements are accessible. Phase retrieval approaches in neutron and X-ray measurements are mathematically motivated in textbooks^1,2^ without revealing the eventual role of event size, possibly as insurance against a solution being found. Phase retrieval methods are highly diverse and the following are just two relevant examples. Multi-wavelength anomalous diffraction (MAD) near elements' absorption edges gives an adjustable reference wave within unit cells for phasing.^1^ This requires multiple X-ray energies, potentially motivating good photon energy resolvability in detectors; it is otherwise the same as conventional X-ray diffraction of von Laue and the Braggs. Neutron and ultrafast X-ray Laue work demonstrates that exceptional source coherence properties are not needed for protein-scale structural dynamics diffraction studies,^6,66,67^ but again motivate a capacity for photon energy resolvability in detector arrays. Those features are a central theme of this work.

X-ray sources including synchrotrons were originally motivated in terms of brilliance largely because it allows increasingly efficient diffraction through the *ΔE/E = Δθ/*tan *θ* ≈ *(3√2/π)(d/n)^2^(r_0_|F|/v_c_)* ≈ 10^−5^ Darwin^68^ rocking curves of typical monochromator crystals (here *d* is interplanar spacing, *n* is the order of the Bragg reflection, r_0_ is the Thomson scattering length, and *F* is the structure factor for the unit cell of volume *v_c_*).^1^ The idea was that the user may then do as they please in *S(Q,ω)* space, if they can address particular membrane-thin cuts through that space in any representative way, with freedom to *scan θ* and/or *E* when necessary.

We sum up this section with reference to Figure 1. A brilliance-based approach overlooks the alternative of addressing *volumes* of the same space *in parallel* using short pulsed but polychromatic and potentially divergent sources, and using these features to spatially resolve the energy of received quanta *by the detector*. This work's incentives of atomic and small molecule motions that occur on femtosecond timescales^69^ accept ongoing needs for hard radiation phase retrieval, with conventional models and other constraint-based methods. Rather than adding confusion to that problem, its strategy is instead the accumulation of large volumes of ultrafast *S(Q,ω)* space *via* energy dispersive approaches, which very efficiently use the *colour* of individual X-ray photons. Semiconductor arrays have for many years offered a powerful opening in this regard. Their capability is now extended in a thermodynamically thorough way, by low temperature thermal X-ray detector arrays.

## NEUTRON–X-RAY OVERLAPS

Without prospects of extreme brilliance sources, the neutron community's approach to the *S(Q,ω)* observations it had fostered and extended^13,14,17,70,71^ took the necessary path. Their needs for accurate low-loss broadband quantum energy measurement were answered by TOF developments,^72–74^ soon aided by cold war pressures and consequently available resources. The same need had no comparable answer for the relatively mature international X-ray community when lasers and synchrotrons were developing. This despite the dawning of some relevant thermal detection technologies,^75–77^ strong awareness of statistical mechanics considerations,^78^ and many examples of pulsed broadband X-ray sources developed before, during, and since that time.^79–82^ This work's suggestion^83^ to combine the ultrafast laser-driven X-rays and low temperature thermal detection was thus built on a heritage^26,84,85^ of neutron structural dynamics studies of molecular,^70,86,87^ crystalline^71,88,89^ and superconducting^90^ systems involving fundamental chemical timescales. A confluence occurred of backgrounds in TOF neutron usage,^84^ time-resolved X-ray diffraction development,^21^ lab-based X-ray source development,^83^ and X-ray detector characterization.^34^ A connection was built from ultrafast laser physics communities to low temperature thermal physics communities by attaining the Fano resolution limit in semiconductor arrays, recognising its physical cause and initiating action to surpass it while knowing the potential. Today it increasingly offers inroads to many known and contemporary ultrafast X-ray developments.^5,9,91,92^

In structural dynamics, neutrons complement X-ray work, especially for studies of light atom, isotope contrast, and magnetic/spin systems. Neutron TOF results are often co-refined or in parallel refined^93^ with X-ray data from tuneably monochromated broadband sources.^85,94^ The lower noise of X-ray data in the latter studies arises from the greater eventual number of detected quanta despite narrow bandpass monochromation (∼Darwin width), showing the value of X-ray brilliance there. From the outset, molecular structure determinations using TOF neutron techniques^4,95^ practiced atomic resolution polychromatic phase retrieval, just as X-ray Laue techniques also did even in the absence of direct quantum wavelength information.^3,6,96^

The high cost of neutron facilities requires instruments to make the most of fluxes that struggle to match what can be provided by simple lab-based ultrafast laser-driven X-ray sources.^81,83^ A great diversity of neutron techniques avoid Darwin-Bragg loss using TOF methods in polychromatic *S(Q,ω)* measurements.^97^ In relative terms, TOF is inapplicable to X-rays because of the essentially fixed (light speed) velocity of X-ray quanta, noting that the narrow phase space utility of refractive and reflective X-ray optics and line gratings severely restricts their application.

Achievements by TOF neutron communities have fully demonstrated the viability of accurately observing large volumes of *S(Q,ω)* space in setups based on energy measurements of individual quanta. That is also the potential opened by combining ultrafast laser driven X-ray sources and low-temperature thermal array detectors, in lab-based ultrafast measurements.^5,83^ Looking beyond individual event measurement, links between neutron and X-ray needs also appear in the very relevant “unfolding” of neutron and X-ray spectra.^98–102^ In these, a combination of prior knowledge and statistics is used to extract spectra from within measurable inner product integrals (typically pileup intensities). While uncertainties do propagate,^103^ the approach is capable of broad applicability. A similar situation applies to Bayesian spectral analyses.^90,104^

## ENERGY DISPERSIVE X-RAY DETECTORS

For polychromatic X-rays, Darwin-Bragg losses are avoidable using energy-dispersive semiconductor array detectors.^105–108^ In favourable cases, these show a Fano/bandgap-limited energy resolution.^34,109^ Depending on the application, that level of resolution can suffice for quantitative spectroscopic identification of elements in samples, and interpretation of polychromatic diffraction data^105–107^ analogous to earlier TOF techniques for neutrons.

Cryogenic microcalorimeter arrays make a deeper appeal to statistical physics and take the long-term scope of such X-ray detectors to a new level.^76,110–112^ In effect they replace the semiconductor bandgap-related Fano energy resolution bound for partial measurement of incident X-ray photons' energy, with a temperature-related bound for their complete measurement. The growing use of cryogenic microcalorimeter arrays for X-ray photon measurement corresponds more directly to the introduction of TOF techniques for neutrons, since both open access to *S(Q,ω)* space using philosophies that permit bypassing the Darwin handicap of Bragg diffraction at high energy resolution. By doing so, they allow gainful examinations of very low radiation levels.

The energy range for thermal detectability spans the full X-ray region as well as the rest of the electromagnetic spectrum and includes the measurement of energetic particles.^111^ In low temperature microcalorimeters, the range from terahertz^113^ to gamma^114^ photon energies may be considered. There it can be broadly stated that at the low energy end, photon wavelengths become larger than the pixel size; while at the high energy end, the cross-section for radiation absorption and opening of new radiation-loss channels become troublesome. Low cross-section requires physically larger absorbers, with reduced pixel density, greater heat capacities, and longer thermal conduction timescales. In the X-ray range, the losses have several potential causes, most notably non-thermalised photoelectron escape^34^ and X-ray fluorescence (XRF).^115^ These lead to partial energy registry by the detector (“spectral redistribution”^116^), in which the observed spectrum may betray other loss mechanisms, too (see, e.g., the inverted Bi absorber L-edges in the low energy tail on page 67 of Ref. 18). Where suitably quantified, the losses can be largely compensated by suitable stripping algorithms.^117^ The desirability of suppressing such losses in microcalorimeters and some ways to do it were identified early.^76^ As radiation energies get higher, more energy escape mechanisms become possible. Those loss channels open up, and the spectral redistribution becomes more complex at the expense of the incident spectrum whose observation is sought.

## THERMAL AND NOISE BOUNDS ON X-RAY ENERGY RESOLUTION

**FIG. 2.**
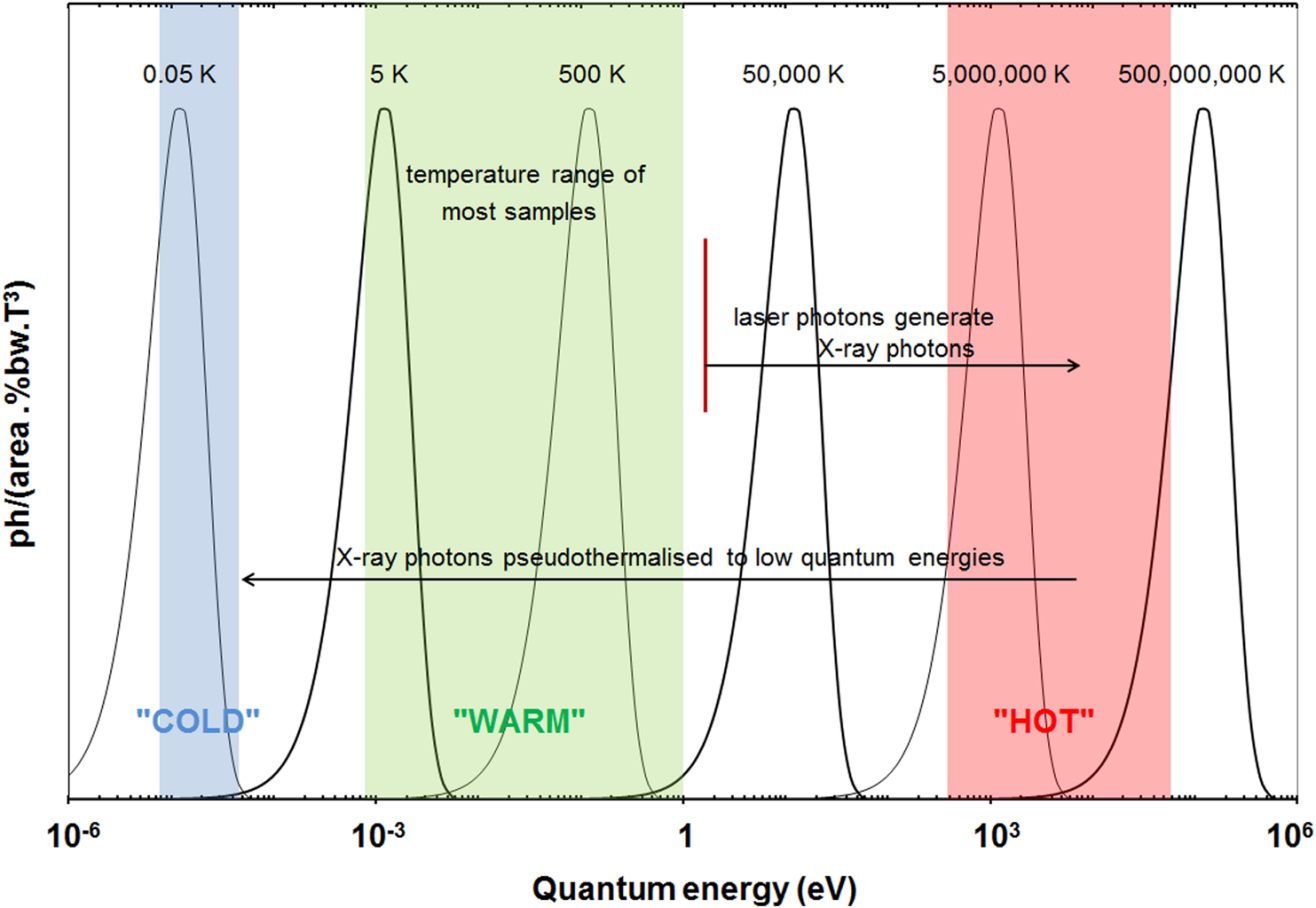
The statistical manipulation of quantum energies is central to our avoidance of Darwin-Bragg losses. A photon may be assembled from lower energy ones (e.g., laser generated X-rays), or broken into lower energy quanta by thermalisation. When heat is measured in a way that includes all the fragmentary quanta, their number places a statistical limit on the accuracy of knowing the parent photon's energy. Planck blackbody distributions allow an idealisation, here on a logarithmic energy scale with Wien maxima spanning equivalent temperatures in experiments to date. Temperatures shift in proportion to the quantum energy, anticipating *ΔE/E ∝ T^½^* and offering a first upper bound on measurement temperatures. For example, *ΔE/E = *10^−4^ for 1 eV resolution of a 10 keV photon (∼5 × 10^7^ K Wien maximum) would need a 10^−8^ effective temperature change for its measurement, i.e., a Wien maximum no greater than 0.5 K. Treatment of thermal flows in calorimetry imposes a stronger temperature dependence, scaling as *(k·T^2^·C)^½^* due to fluctuations at the receiving bath temperature. Convolutions with instrument noise require somewhat lower temperatures still.^111,112^ X-rays' passage through samples, here at relatively ambient temperatures, leaves its *S(Q,ω)* imprint on their ensemble before thermalisation.

Following the theoretical accounting for the photoelectric effect in 1905 (Ref. 118), it was recognised that individual X-ray quanta may be decomposed into a very large number of lower energy excitations, that collectively obey energy conservation. Bolometry is a limiting case and invokes the final thermalisation temperature for the average energy of the eventual excitations (*∼kT*). With sufficient instrument design, the thermal effects from a single X-ray photon are quantitatively measurable in pixels of small size and known location. Two aspects of this are important here. First, it is a zero loss alternative to Bragg diffraction selection for accurately determining the energy *E* of individual X-ray quanta. Second, that accuracy is fundamentally constrained by the detector's temperature. In a naive first-order argument, a number *N_av_ = E/kT* of low energy excitations are generated in an absorber initially at absolute zero (0 K). This number fluctuates statistically as *N_av_*^½^ due to the many combinations of ways to distribute that energy.^119,120^ (An analogous treatment effectively estimates the number and energy of quanta in shot-noise limited radiation measurements.^121^) Applying this somewhat impractical argument to determine the energy of the parent X-ray photon, Poisson statistics applied to the limiting case of Planck distributions in Figure 2 then suggest a limiting X-ray energy resolution *vs.* temperature scaling as *ΔΕ/Ε ∝ T^½^*. Corresponding X-ray measurement temperatures are necessarily very low. In practice, energy flow to a reservoir at finite temperature is indicated in order to allow physical measurement, which in this context motivates calorimetry. Calorimetry leads to a still lower thermodynamic bound with a stronger temperature dependency^76,77,111^ according to thermal fluctuations of magnitude *(k·T^2^·C)^½^* at the temperature of the receiving bath.^120,122^

Pixels' absorber materials and their physical dimensions are constrained by the stopping power for incident radiation and the need to avoid energy trapping and loss mechanisms (i.e., non-thermalisation channels). Thermalisation must occur on timescales briefer than the readout. Moreover, noise is invariably contributed by other aspects of the apparatus used to convey the measured thermal signal. That leads to ongoing efforts to witness, account for, and eventually remedy any excess noise.^123–128^ Noise sources generally have spectral dependence^129,130^ which further constrains the eventual measurement bandwidth. Signal transfer typically involves nonlinear amplifiers (e.g., superconducting quantum interference devices (SQUIDs) in transition edge sensor (TES) systems^112^) and multiplexing arrangements, with the various noise sources convolved at the output. There is consequently a high art in the design procedure for any such detector. Different approaches, including several technologies described in Ref. 111 and perhaps one day the optical damping suggested in Ref. 5, are developing in parallel, each with corresponding noise considerations.

Figure 2 and the approach above to a “first-guess” upper bound of detection temperature already make it clear that when in a single photon mode, a comparable *ΔE/E* measurement of lower photon energies will require lower temperatures. Base temperatures in the range ∼0.05–0.1 K have often been used in ∼1–50 keV X-ray work to date. When in pileup mode, spectral information may be extractable through unfolding procedures, as mentioned earlier. This approach can have value when photon energies are too low to make discernible individual contributions, while their pileup does not exceed the dynamic range of readout components. For visible photons and much of the range below it, photons can be dispersed without loss using line gratings or on the basis of refractive dispersion (prisms). In the X-ray range, the restrictive capabilities of refractive, reflective, and line grating optics give low temperature thermal measurements a unique role.

## PROSPECTUS IN CONTEMPORARY CONTEXTS

For X-rays, the key appeals of cryogenic microcalorimetry as an alternative to Bragg diffraction are that it does not similarly constrain instrument geometry and topologies. Like TOF for neutrons, it is free from any Darwin-Bragg throughput loss in broadband measurements, while not having a Fano resolution limit imposed by semiconductor bandgaps.^34,109,131^ Together, these features can be crucial when designing instruments around low-flux X-ray sources. Historically, chemical application developments around lab-based ultrafast X-ray sources were impeded by the Darwin-Bragg losses incurred in diffracting samples and/or diffractive analyser optics.^132–135^ X-ray microcalorimeters eliminate that constraint. Beyond efficiently revealing a sample's structure and dynamics *via* its detectable *S(Q,ω)* function, the development of cryogenic X-ray microcalorimeter detectors is timely in an evolutionary sense. This is because in-house hard X-ray capabilities driven by ultrafast lasers have been very extensively recognised and developed in recent decades.^9,81,135–137^ While temporally remarkable and now fairly widespread in academic communities, many variants give broadband and low brilliance X-ray output, in some cases with tuneable polychromaticity, divergence, and polarization.^9^ These X-ray application development environments are powerfully motivated when they are enabled to retrieve atomic and molecular structure dynamics information. For X-rays, low temperature microcalorimeters are currently the only way to explore *S(Q,ω)* at high resolution while avoiding Darwin-Bragg losses. They enable diverse X-ray studies^138,139^ that naturally include usage at large facilities.^140–142^

## THE CURRENT STATE OF PLAY

Cryogenic microcalorimeters are in a rapid stage of applied development. There has been practical awareness of the enormous burden of Darwin-Bragg losses, the handicaps of X-ray brilliance, and recognition of the fundamental need for low temperature thermal detection to overcome the Fano-limit of bandgap-based detectors. Equally critical were insights to the parallel roles of X-ray microcalorimeter arrays and TOF for neutrons and the resulting scope for in-house and ultrafast *S(Q,ω)* measurements when combined with polychromatic laser-driven X-ray sources. Putting all those things together, work instigated^83^ arrangements combining a very simple laser driven hard X-ray source and a first generation of cryogenic microcalorimeter arrays, to raise awareness of the potential that opens up when avoiding Darwin-Bragg losses in ultrafast X-ray work.^18,19,142^ The suggestion's origins in both neutron and X-ray structural dynamics made the combination's long-term scope for *S(Q,ω)* measurement diversification implicit from the outset. Thus it targeted the opening of lab-based ultrafast broadband X-ray applications in a broad sense^5^ despite prototypical constraints.

The use of low-T thermal detectors in the ultrafast field is to date constrained by the number of available pixels and their response time, such that their ongoing development in this context is mandatory. Measurements using them have so far had access to systems containing only a few tens or hundreds of pixels. Nevertheless, lab-based hard X-ray pump-probe chemical studies are increasingly being realised, with the potential for temporal resolution corresponding to the lasers' properties. They were initiated to provide structural input to contemporary excited state studies,^143,144^ including the observation of coherent control experiments^69,145–147^ on the laboratory scale.^5,83^ The basic underlying X-ray techniques are widespread at synchrotrons and other large scale facilities where they have matured for a steady state and routine use, using traditional Bragg-diffraction optics. However, there they do not indicate in-house capability, while often operating near conceptual performance limits, at the same time with many technical or even social^148^ constraints on ultrafast pump-probe development. By comparison, ultrafast electron microscopy approaches that make the most of strong electron-atom interaction cross sections and avoid space-charge pulse broadening of electron pulses have become commercially available^149,150^ following the pioneering work of Zewail.^151–154^ Angular resolved photoelectron TOF is also the basis of important contemporary efficiency developments in X-ray spectroscopy.^155,156^ There is a common ground, including at the level of eventual samples.^157,158^

**FIG. 3.**
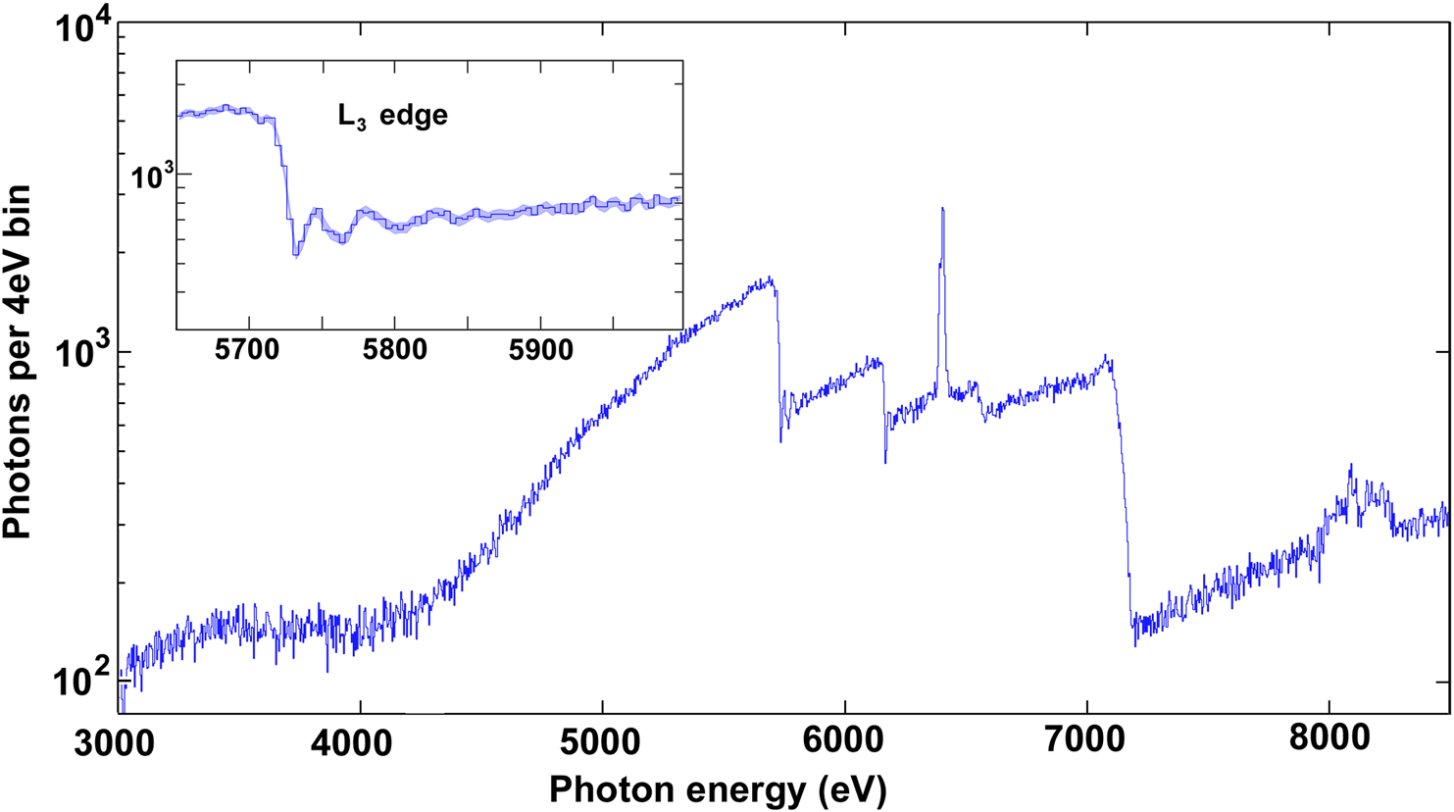
A prototypical broadband X-ray transmission spectrum, from two absorption lengths of CeCl_3_ (above L_3_ edge) on filter paper. A Fe_2_O_3_ three absorption length (above ∼7.1 keV Fe K edge) X-ray filter on the detector physically windowed the spectral ROI and introduced the K_α_ XRF doublet lines at ∼6.4 keV. Distance and laser parameters^34^ were also used to limit the photon arrival rate. These in-house source^83^ and detector^110^ measurements use the philosophy of Figure 2 to avoid the losses described by Figure 1. When measured in 2013, these were the first L-edges observed using a laser-driven X-ray and low-T microcalorimeter combination, other than the ∼13–17 keV Bi L-edges of the 2.5 *μ*m thick absorbers themselves.

The first prototypical realisation^18^ of any ultrafast laser-driven broadband X-ray source plus low-temperature thermal detector combination^19^ was used to record Figure 3. In variants of the particular detector systems used so far,^110^ individual event record times of ∼10 ms have been used to obtain a good energy resolution. That should be compared with the 1 ms pulse repetition period for a typical 1 kHz laser capable of both generating hard X-rays and optically stimulating a sample. It governs the rate for data acquisition, where a spatiotemporally Poissonian photon arrival rate may be assumed (see, e.g., page 61 in Ref. 18). In that particular situation, the consequence is optimal data rates typically a few tens of counts per second per pixel.^19^ Distances, laser parameters, and spectral filter materials were adjusted to achieve the desired spectrum and flux on the detector. Poissonian counting noise is seen due to the very few (∼30) pixels available in this few-hour measurement. Artefacts are also evident and include partial registry intensity below the filters' soft low cutoff at ∼4 keV; peaks above ∼8 keV due to component saturation for some detector pixels; and potential spectral inaccuracies due to the provisional pixel linearisation/calibration/co-addition algorithms used here. The absorbers' Bi L-edges are excluded from depiction at their somewhat higher energies in this particular detector system^18^ by SQUID response nonlinearity, being typically rejected in Microcalorimeter Analysis Software Suite (MASS) filtering (below). Oscillations above the Ce L-edges relate to local electronics and bonding,^19,159,160^ while XRF fine structure offers simultaneous ultrafast chemical and magnetic spin structure.^139,161^ While prototypical, results such as in Figure 3 and their objective of time-resolved work have helped to stimulate further development by our groups in a detector development environment,^158,162^ building on an appreciation of temporal prepulse symptoms^121^ to motivate a dedicated and superior laser system. This has led to successful recent ultrafast pump-probe measurements in X-ray emission spectroscopy (XES)^163^ and X-ray absorption spectroscopy (XAS).^164^

In transmission XAS measurements such as Figure 3, filters' thickness and absorption edges have been calculated to arrange a spectral ROI on extremely broadband isotropic laser-plasma hard X-ray sources. This is sometimes done together with polycapillary optics^158,163,164^ to manipulate polychromatic divergence towards sample and/or detector surfaces. They and other X-ray optics can be helpful to suppress high energy photons beyond what the quantum efficiency of the detector's finite absorber thickness also does; varying parameters of the X-ray generation laser and target environment can be used to similar effect.^34^ The same X-ray optics can compromise spectral and temporal aspects,^165^ issues that together with technique diversification will lead to thermal detectors' conjunction with other varieties of ultrafast laser-driven X-ray sources. Prototyping challenges were eased using deliberately simple and versatile water jet-based^166,167^ ultrafast X-ray sources^83,136^ and their target chambers.^168^ Their simplicity enabled many further characterization,^34^ mechanistic^169^ and temporal contrast^121^ examinations.

**FIG. 4.**
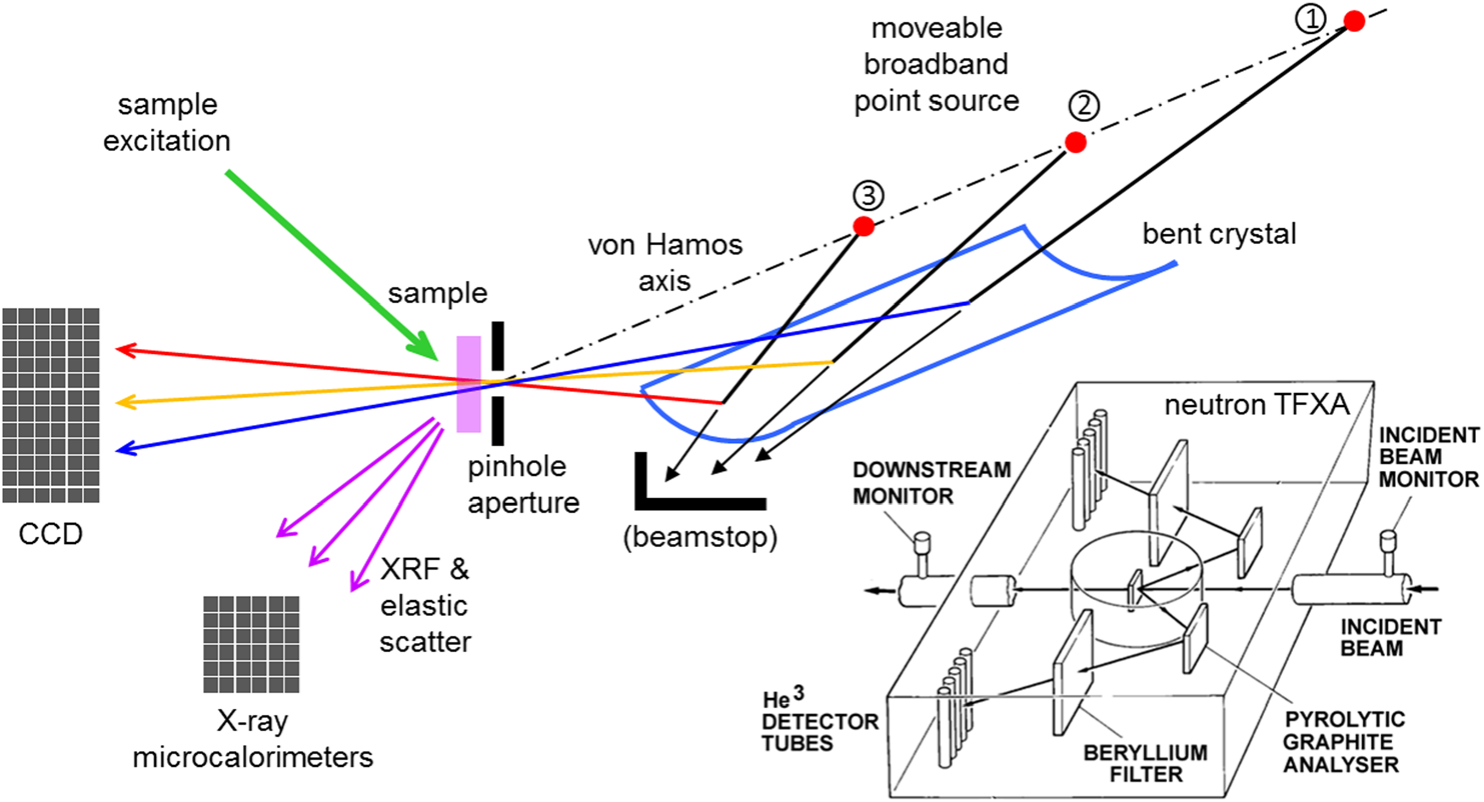
RIXS proposal: A sample point region of continuously variable energy X-rays is arranged, here inverting a von Hamos scheme from a divergent polychromatic source. The source is repositioned along the axis from position ①–③ to give pinhole transmission of different photon energies. The X-ray energy incident on the sample is accurately known from distances and d-spacing in the spectrometer, supplemented by transmitted beam positions on the CCD. Elastic scatter from the sample and its environment assists calibration of the microcalorimeters, which otherwise also detect the XRF and its potentially resonant inelastic components as the incident energy is tuned. The inset at bottom right sketches the former time focussed crystal analyser (TFXA) instrument at the ISIS-I pulsed neutron source, a conceptually similar combination of diffraction and TOF for a highly successful inelastic neutron scattering spectroscopy instrument (reproduced with kind permission of the ISIS facility).^84,174,175^

Analogous broadband results had earlier been measured using temporally stochastic beta-decaying elements in carefully chosen samples.^170^ As with semiconductor detectors, X-ray emission examinations have an extended history in the development of X-ray microcalorimeters,^77,111^ yet demonstrations using fast ions^139,171^ and at chemical resolution using lab-based femtosecond laser-driven X-rays^142,172^ are now greatly expanding the prospects. Resonant inelastic X-ray scattering (RIXS) measurements^161,173^ are also a potential target. Figure 4 shows one potential scheme to access lab-based ultrafast RIXS measurements. Like its TOF neutron predecessors for inelastic neutron scattering,^26,84,174,175^ it seeks to combine the microcalorimeters' strengths of high resolution lossless detection with the narrow bandpass capabilities of Bragg diffraction-based optics.^176–179^ The scheme suggested in the figure has the feature that the sample-incident monochromatic X-ray energy depends on the distance from the polychromatic source to the sample (*via* the crystal). Generally that is undesirable in ultrafast work and for mechanical alignment reasons. In practice, however, an optical pathlength that compensates for the change of temporal coincidence can be arranged when necessary. Depending on temporal needs and experimental particulars, it may be preferable to optically pump the sample through the X-ray pinhole. The arrangement is simple enough that it or variants could be explored using existing water jet laser plasma and microcalorimeter setups.

To date, this collaboration has used TES-based detectors developed in NIST's Quantum Sensors group.^18,110^ Hardware and software stabilises four stages of refrigeration, establishes and holds optimum TES bias voltages, and enables pixel readout through three levels of SQUID-based multiplexing feedback electronics. The TES sensors are voltage biased to improve response time and hold an approximately constant operating point on the superconducting transition, by a dynamic balance of ohmic heating and X-ray power from the thermally linked absorbers (electrothermal feedback). In the scheme used so far, current through the TES passes through one of two thin-film coils coupled to a SQUID, whose output is monitored and nulled in a feedback loop that drives the other coil via room temperature circuits. The feedback and error signals are monitored, and an optimally chosen linear combination is saved as a raw data trace. During setup, each pixel's feedback loop requires a proportional-integral-derivative (PID) optimisation of stability and noise. Manual operation has been routinely done but is nontrivial and tedious with many pixels, while digital integration has allowed increasing automation as software developed. Meanwhile, different front end and multiplexing schemes are explored and coming into use (time division,^110^ code division,^180^ and frequency division^181^ multiplexing). X-ray events arrive as multichannel oscilloscope traces, whose amplitudes and shapes relate nonlinearly to the energy of X-ray photons. Each pixel has a different nonlinearity. Potential for on-the-fly iterative data interpretation^182^ is plain to see, but an immediate priority is to store the traces, which heavily commits computer memory bandwidth.

The Microcalorimeter Analysis Software Suite (MASS) is a large and growing body of Python codes to extract X-ray photon energies from the saved traces; no subsequent X-ray data analysis is currently possible without first becoming intimately familiar with operations using MASS. It allows for many aspects of event parameter estimation,^183^ filtering^184,185^ and calibration,^186^ compensation of instrumentation artefacts and drifts,^187^ partial pileup of electronically distinguishable events,^188^ pixel coaddition, and general presentations and manipulations of the multidimensional data. Detector hardware, its software control, and MASS evolve together as numbers of pixels, readout circuits, data rates, and energy resolutions all find new compromises. A working knowledge of the existing detector hardware implies also an understanding of low temperature and condensed matter physics with skills in practical electronics. To aid new users from other backgrounds, these various needs are gradually being alleviated.

The introduction of X-ray microcalorimeters to lab-based ultrafast X-ray science can enormously reduce the X-ray flux needed for measurements, being attractive for the protection of both personnel and samples. X-ray spectroscopic and diffraction applications will generally seek to exploit the factor of ∼10^5^ that has been won for in-house explorations of *S(Q,ω)* space. Significant lead times for detector developments mean that existing systems have been applied in contexts where they were not yet expected to be competitive with major user facilities. The diversity of low temperature thermal detector technologies,^111^ their increasing commercialisation, and recognition of key application fields^112^ will influence this situation. A link has been made to major application fields. Variants will spawn as detector capabilities improve, and as source development communities become aware of what has been opened. The situation is transformational for molecular structure dynamics studies using X-rays on the scale of the home laboratory and on ultrafast timescales.

The applied future of ultrafast laser-driven X-ray sources clearly demands parallel investments in detector hardware, and always has. Among other things, hardware investments seek a greater number, fill factor, density, and dynamic range of rapidly thermalising absorber “pixels” at low temperature, and high bandwidth low noise instrumentation through which to accurately measure and analyse signals. This will aid ultrafast energy-resolving polychromatic *S(Q,ω)* measurements where spectral resolution and higher detector stopping power is increasingly desirable in what are otherwise direct CCD^107^ and higher-Z semiconductor arrays.^189^ (For broadband ultrafast work where photons are valuable at the same time that some pileup is inevitable and potentially still useful, systems obviously must avoid the frustration of front end digital discriminator circuits.^190,191^) At the correspondingly deep level of solid state quantum physics, studies of thermal properties central to these efforts are a contemporary and beautiful field.^192^ They are among the research interests of the low temperature expert groups that have collectively enabled this work.^112,193,194^

## CONCLUSION

A thermal physics viewpoint of X-ray generation at high temperatures and detection at low temperatures allows powerful new approaches to material examinations. Cryogenic microcalorimeters offer for X-rays what time of flight techniques do for thermal neutron detection.^5,83^ Broad application scope arises from efficient experiment topologies in both cases, that avoid the potentially ∼10^−5^ Darwin throughput associated with Bragg diffraction. In both cases, the alternative to Bragg diffraction additionally gives opportunities to complement it, enabling new and highly efficient X-ray instrumentation designs. The energy of femtosecond optical laser photons has been used to generate broadband hard X-ray photons in lab-scale ultrafast development-friendly experiments; these X-ray photons have been passed through samples for the purpose of *S(Q,ω)* examinations including in pump-probe studies, and quantitatively disintegrated on an individual basis to very low energy quanta by pseudo-thermalisation in prototypical detector systems. By altering and examining the colour of photons in this way, we have demonstrated a central role for statistical thermodynamics in unprecedented realisations of lab-based ultrafast X-ray instrument design. Our initiation of this approach dramatically extends the scope for in-house chemical X-ray structure dynamics examinations using ultrafast and low brilliance X-ray sources, a field that meanwhile^21,22,195^ became popular at high brilliance synchrotron and XFEL facilities.
